# Impact of Life-Cycle Variation on Feeding System Musculature in Caudata

**DOI:** 10.1093/icb/icag040

**Published:** 2026-05-07

**Authors:** Morgane Taillades, Isabelle Toussaint-Lardé, Vivien Louppe, Morgane Fournier, Mark Mandica, Anthony Herrel, Anne-Claire Fabre

**Affiliations:** Mécanismes Adaptatifs et Evolution, UMR 7179, Muséum national d’Histoire naturelle CNRS, Paris, 75005, France; Institute of Ecology and Evolution, Universität Bern, Bern 3005, Switzerland; Naturhistorisches Museum Bern, Bern 3005, Switzerland; Department of Biology, Evolutionary Morphology of Vertebrates, Ghent University, Ghent 9000, Belgium; Mécanismes Adaptatifs et Evolution, UMR 7179, Muséum national d’Histoire naturelle CNRS, Paris, 75005, France; Institute of Ecology and Evolution, Universität Bern, Bern 3005, Switzerland; Naturhistorisches Museum Bern, Bern 3005, Switzerland; Institute of Ecology and Evolution, Universität Bern, Bern 3005, Switzerland; Naturhistorisches Museum Bern, Bern 3005, Switzerland; Institute of Ecology and Evolution, Universität Bern, Bern 3005, Switzerland; Naturhistorisches Museum Bern, Bern 3005, Switzerland; Amphibian Foundation, Atlanta, GA 30342, USA; Mécanismes Adaptatifs et Evolution, UMR 7179, Muséum national d’Histoire naturelle CNRS, Paris, 75005, France; Naturhistorisches Museum Bern, Bern 3005, Switzerland; Department of Biology, Evolutionary Morphology of Vertebrates, Ghent University, Ghent 9000, Belgium; Department of Biology, University of Antwerp, Wilrijk 2610, Belgium; Institute of Ecology and Evolution, Universität Bern, Bern 3005, Switzerland; Naturhistorisches Museum Bern, Bern 3005, Switzerland; Department of Life Sciences, Natural History Museum, London SW7 5BD, UK

## Abstract

Caudata (salamanders and newts) exhibit considerable diversity in terms of ecology, life cycle, morphology, and behavior, ranging from species with complete metamorphosis to those with facultative or complete loss of metamorphosis. These developmental differences are often tightly linked to ecological transitions and morphological transformations, influencing how salamanders exploit habitats, access resources, and feed during their lifetime. While skeletal transformations have been widely studied, the impact on cranial musculature remains poorly understood. This study explores how life-cycle variation and associated ecological transitions and morphological transformations affect the architecture of feeding muscles in salamanders. We conducted dissections of the feeding system in 25 salamander species representing different life cycles, ecological transitions, and morphological transformations. We quantified muscle volume and physiological cross-sectional area (PCSA) functional muscle group of the jaw and hyoid muscles. Our results revealed a differentiation in cranial musculature based on different ecological strategies, and that other factors, such as head size, play a prominent role in shaping muscle architecture. We identified consistent patterns associated with whether individuals undergo an ecological transition, experience a morphological transformation, and with their adult habitat use, suggesting that ecological context imposes functional constraints on the muscular organization of the feeding system. These findings suggest that life history, ecological, and developmental strategies impose constraints that influence the muscular organization of the jaw and the hyoid apparatus. Future work should broaden taxonomic sampling, integrate bone and muscular traits together, and examine the evolutionary pathways by which life-cycle variation interacts with functional morphology in Caudata.

## Introduction

Metamorphosis is highly prevalent among amphibians (Bishop et al. 2006) and involves fundamental morphological and physiological transformations, often associated with habitat shifts. This habitat transition coupled with developmental complexity drives profound structural and functional changes, as individuals transform from an aquatic larva to a terrestrial adult (Reiss 2002). However, to disentangle the respective influences of ecological transitions and developmental complexity on morphological and functional changes, it is essential to study organisms that exhibit a wide range of life-cycle types, from complex to simple, and that differ in the nature of their ecological transitions. In this context, salamanders and newts (Caudata) are a great model system as they display a tremendous variation in life-cycle types (Bonett and Blair 2017; Wake and Koo 2018; Fabre et al. 2020; Bonett and Ledbetter 2022; Louppe et al. 2025). Some are biphasic, undergoing complete metamorphosis, sometimes with an ecological transition (water to land). In the case of biphasic species that undergo an ecological transition, two major functional changes occur: locomotor shifts from swimming to walking and feeding changes from suspension or suction feeding to jaw or tongue prehension (Reiss 2002; Herrel et al. 2019). A few species are even multiphasic, encountering a second metamorphosis during their adulthood, with the adult being primarily terrestrial but becoming seasonally aquatic during the breeding season (Deban and Wake 2000a, 2000b). In these species, both ecological transitions (from water to land and *vice versa*) and morphological transformations occur several times during the lifetime of the animal. In contrast, other species exhibit paedomorphosis, including for example, several *Ambystoma* species (e.g., *Ambystoma mexicanum, Ambystoma andersoni*), the olm (*Proteus anguinus*), and species of the family Sirenidae, where adults retain larval traits, such as external gills, even after reaching sexual maturity (Gardner Lynn 1961; Denoël 2002; Bonett and Ledbetter 2022). These species exhibit incomplete metamorphosis without undergoing an ecological transition, remaining fully aquatic throughout their life cycle. In addition, several species are direct developers [such as most terrestrial lungless salamanders (e.g., *Plethodon*)], and individuals hatch as fully formed juveniles. This developmental mode enables emancipation from aquatic environments by eliminating the free-living larval stage (Wake and Hanken 1996), with a complete metamorphosis occurring within the egg (Gardner Lynn 1961). Consequently, these species undergo no ecological transition and persist as terrestrial organisms throughout their lifespan. Other species are viviparous (Gardner Lynn 1961) (e.g., the Alpine salamander (*Salamandra atra*), the Fire salamander (*Salamandra salamandra*), and the Lycian salamander (*Lyciasalamandra antalyana*)), with the mother giving birth either to a fully developed newborn (i.e., pueriparous) or to a larval stage in water (i.e., larviparous). Finally, a few species exhibit a facultative life cycle, in which developmental modes vary within the same species and among different populations, likely in response to variation in environmental conditions (Denoël and Whiteman 2007). For instance, in certain species [e.g., the Alpine newt (*Ichthyosaura alpestris*), the Tiger salamander (*Ambystoma tigrinum*), and the Eastern newt (*Notophtalmus viridescens*)], some populations are multiphasic or paedomorphic. Additional examples of facultative life cycles occur in fire salamanders, where populations may be either pueriparous or larviparous (Whiteman 1994; Denoël and Joly 2001; Mulder et al. 2022).

The diversity of life cycles and associated metamorphosis (from partial to complete metamorphosis) have impacted morphological diversity in Caudata probably contributing to their ecological diversification across a wide range of environments and feeding modes (e.g., Fabre et al. 2020; Louppe et al. 2025). As such, they can be terrestrial, semi-aquatic, or fully aquatic, and may even be seasonally aquatic, returning to water for breeding (Deban and Wake 2000a, 2000b). Both developmental complexity and ecological transitions involve significant anatomical modifications, affecting both the skeleton and muscles (Ziermann and Diogo 2013). Thus, the constraints differ between larvae, juveniles, and adults, as their habitats and developmental stages impose distinct challenges (Moran 1994). Several studies have shown that life-cycle variation and associated ecological transition have an effect on the morphological pattern of the bony structures of different parts of the body (e.g., limbs: Ledbetter and Bonett 2019; vertebral column: Bonett and Blair 2017; cranium: Fabre et al. 2020; mandible: Louppe et al. 2025). Additionally, numerous studies investigated the functional impact of the variation of feeding mode depending on the habitat occupied by the animal. While most of the studies focus on the impact of metamorphosis types and associated ecological transitions in bone external morphology or function (kinematics of feeding or moving), less attention has been given to feeding muscles. Several studies have provided information on the musculature of Caudata (Krogh and Tanner 1972; Lauder and Bradley Shaffer 1985; Bauer 1997; Ziermann and Diogo 2013), but most of them have concentrated on a single taxon or a few taxa (further relevant studies are summarized in Supplementary Table S1). As such, a comparative analysis investigating the muscles of the feeding system depending on morphological transformations and associated ecological transitions in Caudata is currently lacking.

Here, we study the impact of the completeness of metamorphosis and the associated ecological transitions on the myology of the head system in Caudata. The head is an interesting structure due to its ontogenetic and functional complexity. It houses and protects the principal sensory organs and the brain, and it plays a crucial role in acquiring nutrients necessary for survival. Consequently, feeding muscles may be influenced by shifts in feeding strategy during ecological transitions throughout ontogeny, as well as by the degree of metamorphosis. We hypothesize that (i) individuals that experience an ecological transition from aquatic to terrestrial environments will display significant differences in muscle architecture, particularly in the hyoid and jaw musculature, compared to other that remain in a single environment throughout their life cycle (e.g., fully aquatic paedomorphic salamanders); (ii) developmental trajectories involving a complete post–hatching metamorphosis are expected to result in muscle architectures that differ from those in individuals whose juvenile stages resemble the adult form; (iii) adult habitat is expected to correlate with muscle architecture: predominantly aquatic adults should display more developed hyoid musculature, terrestrial adults should show more robust jaw adductors, and adults occupying intermediate or semi–aquatic habitats are predicted to exhibit mixed or transitional muscular traits.

## Material and methods

### Specimens

**Fig. 1:**
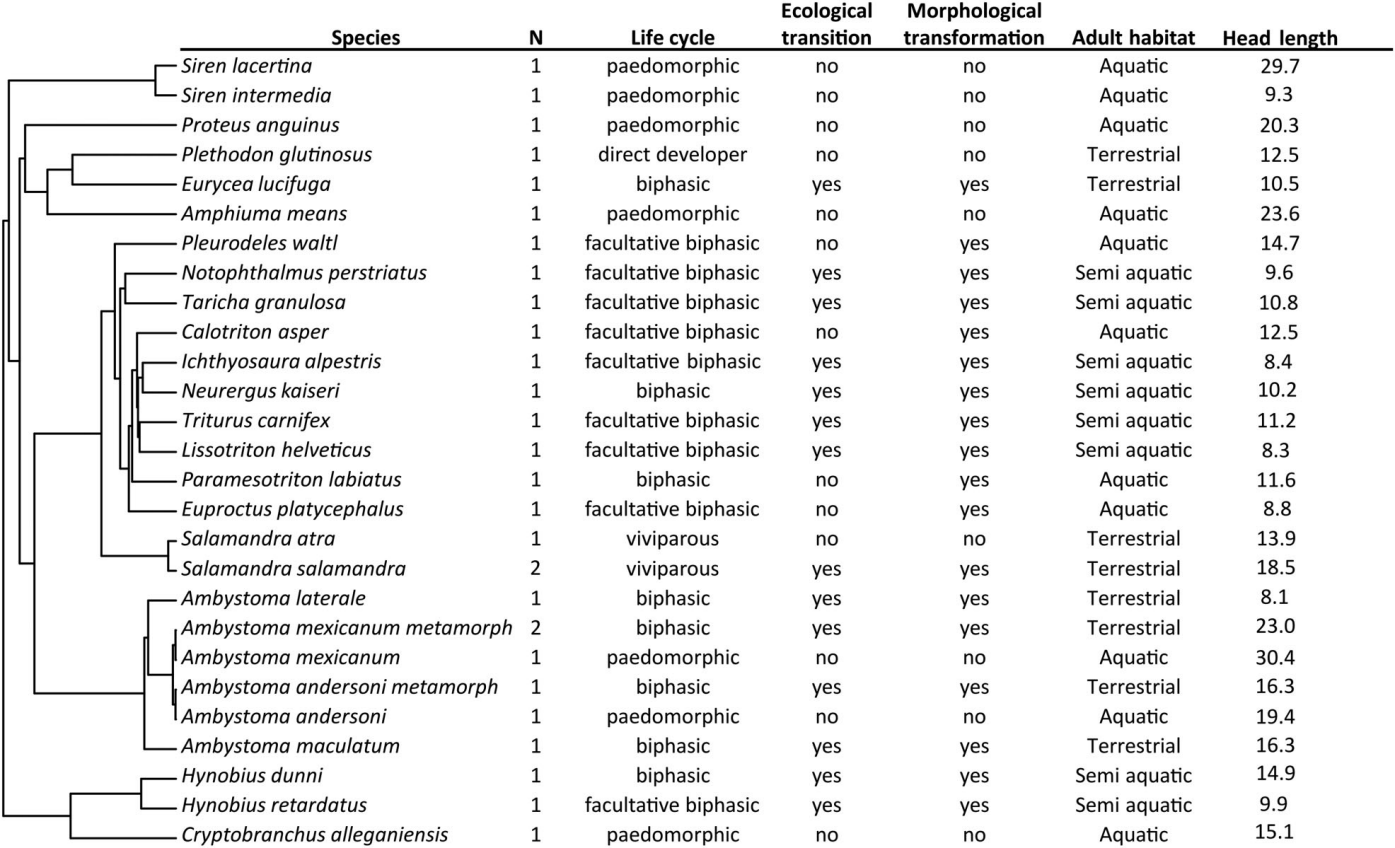
Phylogeny of the species and subspecies studied in this research. Presented, in order, are the species name, the number of specimens dissected for each species, their life cycle, whether or not an ecological transition occurred, whether or not a morphological transformation occurred, the type of habitat used by the adult and the head length in mm.

In this study, 25 species of Caudata were dissected (Fig. 1). All the specimens came from the personal collections of Anthony Herrel and Mark Mandica, as well as from the Naturhistorisches Museum in Bern (NMBE). The specimens were preserved in 70% or 75% ethanol. Each species is represented by at least one adult specimen (for *Ambystoma mexicanum* and *Ambystoma andersoni*, two specimens were dissected: one metamorphosed and one paedomorphic specimen). Because we could not obtain both sexes for each species, our dataset includes whichever specimens were available for destructive dissection. This potential source of variability should therefore be kept in mind when interpreting the results. The list of all the specimens used in this study is available in Supplementary Table S2. The head length (HL) was measured using a digital caliper from the back of the skull, corresponding to the posterior part of the parietal (at the suture with the occipito-otic), to the tip of the snout.

#### Dissection, nomenclature, and muscle properties

All the muscles of the head required during feeding were removed one by one. Jaw muscles were removed unilaterally from each specimen, and hyoid muscles were removed unilaterally when possible. However, some hyolingual muscles were removed bilaterally due to technical constraints related to specimen size or preservation. In these cases, the combined muscle mass was divided by two. Thus, only one side was considered in subsequent analyses. When necessary, a binocular microscope was used (Leica M80). Many synonyms exist in the literature. Supplementary Table S3 presents synonyms found in the anatomical articles reviewed for this study.

Jaw muscles were separated into two functional groups: the muscles involved in opening and closing the mouth respectively.

**Fig. 2:**
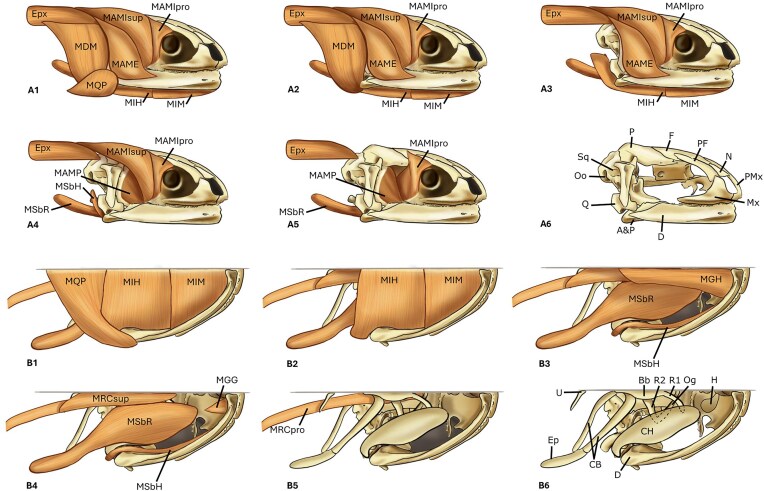
Reconstruction of the head musculature in a hypothetical salamander, illustrating general anatomical organization. This is a schematic drawing which can be applied for non-paedomorphic forms. Epx (Epaxial muscle)(A) Lateral view dissection; (A1) after removal of the skin; (A2) after removal of MQP (muscle quadratopectoralis); (A3) after removal of MDM (muscle depressor mandibulae); (A4) after removal of MAME (muscle adductor mandibulae externus), MIH (muscle interhyoideus) and MIM (muscle intermandibularis); (A5) after removal of MSbH (muscle subhyoideus) and MAMIsup (muscle adductor mandibulae internus superficialis); (A6) skull only. (B) Ventral view, the grey line represents bilateral symmetry; (B1) after removal of the skin; (B2) after removal of MQP (muscle quadratopectoralis); (B3) after removal of MIH (muscle interhyoideus) and MIM (muscle intermandibularis); (B4) after removal of MGH (muscle geniohyoideus); (B5) after removal of MGG (muscle genioglossus), MSbH (muscle subhyoideus), MSbR (muscle subarcualis rectus) and MRCsup (muscle rectus cervicis superficialis); (B6) skull and hyoid. Bones abreviations: A&P (Angular and Prearticular); Bb (Basibranchial); CB (Ceratobranchial); CH (Ceratohyal); D (Dentary); Ep (Epibranchial); F (Frontal); H (Hypohyal); Mx (Maxilla); N (Nasal); Og (Otoglossal); Oo (Occipito-otic); P (Parietal); PF (Prefrontal); PMx (Premaxilla); Q (Quadrate); R1 (First radial); R2 (Second radial); Sq (Squamosal); U (Urohyal).

Mouth opening muscles:

- *Musculus depressor mandibulae* (MDM) (Fig. 2, A2) is sometimes divided into two parts: an anterior and a posterior part (Deban and Wake 2000a). It originates on the posterior face of the squamosal and the quadrate (Deban and Wake 2000a) and inserts at the posterodorsal end of the articular and prearticular (Hinderstein 1971; Lorenz Elwood and Cundall 1994).

Mouth closing muscles:

These muscles are often referred to as “levator” or “adductor” muscles in the literature.

- *Musculus adductor mandibulae externus* (MAME) (Fig. 2, A3) originates on the dorsal surface of the squamosal and the quadrate (Iordansky 2010; Matsumoto et al. 2024) and inserts on the prearticular and the dentary of the mandible (Deban and Wake 2000a).
- *Musculus adductor mandibulae internus* (MAMI) is sometimes divided into a superficial (MAMIsup) (Fig. 2, A4) and a deep (MAMIpro) (Fig. 2, A5) portion (Heiss, 2016). The superficial portion originates on the rostral face of the atlas ridge (Deban and Wake 2000a; Deban and Richardson 2017), while the deep portion originates on the lateral border of the frontal and parietal bones (Hinderstein 1971; Krogh and Tanner 1972). Both portions insert on the quadratomandibular ligament at the coronoid process (Iordansky 2010; Deban and Richardson 2017).
- *Musculus adductor mandibulae posterior* (MAMP) (Fig. 2, A5) is usually small and poorly differentiated (Carroll and Holmes 1980). It originates from the anterior part of the squamosal and quadrate and inserts mainly on the dorsal and posteromedial surfaces of the dentary and the coronoid process (Carroll and Holmes 1980; Lorenz Elwood and Cundall 1994).

Regarding the hyoid muscles, they can be separated into three groups: the levators, protractors, and retractors of the hyobranchial system.

Hyobranchial levator muscles:

Except for the *Muscle geniohyoideus* (MGH), all the muscles presented in this section are thin and composed of a single layer of fibers, located just under the skin.

- *Musculus quadratopectoralis* (MQP) (Fig. 2, B1) is derived from the larval musculus interhyoideus posterior (Hinderstein 1971). When present, it originates from the lateral surface of the ventral edge of the quadrate (Deban and Richardson 2017). The fibers run ventrally to insert on the skin of the gular fold and the medial aponeurosis (Larsen and Guthrie 1975).
- *Musculus intermandibularis* (MIM) (Fig. 2, B1, B2) is often divided into two distinct heads: a smaller *intermandibularis anterior* at the tip of the mandible, and the larger *intermandibularis posterior* (Deban and Wake 2000a). They originate medially from the dentary (Heiss et al. 2016) with fibers running medially and ventrally to insert on the medial aponeurosis (Heiss et al. 2016).
- *Musculus interhyoideus* (MIH) (Fig. 2, B1, B2) shares its origin with the *m. intermandibularis posterior* on the quadrate and the posterior corner of the ceratohyal, inserting on the medial aponeurosis (Larsen and Guthrie 1975).
- *Musculus geniohyoideus* (MGH) (Fig. 2, B3) originates on the urohyal and inserts at the mandibular symphysis (Deban and Wake 2000a).

Hyobranchial protractor muscles:

- Musculus geniohyoideus: the same muscle as described above.
- *Muscle subarcualis rectus* (MSbR) (Fig. 2, B4) originates on the ventral face of the ceratohyal and inserts at the end of the first epibranchial (Deban and Wake 2000a).
- *Musculus subhyoideus* (MSbH) (Fig. 2, B3), when present, it is a small muscle that originates at the posterior end of the ceratohyal and inserts anteriorly on the dorsal fascia of the m. adductor mandibulae internus profundus or on the mandible near the symphysis (Deban and Wake 2000a; Krogh and Tanner 1972; Heiss et al. 2016;).
- *Musculus branchiohyoideus* (MBH) is a larval muscle (only present here for the neotenic forms). When present, it originates on the ceratobranchial and inserts on the ceratohyal and hypohyal.
- *Musculus genioglossus* (MGG) (Fig. 2, B4) is a small muscle located at the tip of the mandible. It originates on the mandibular symphysis and the lingual surface of the dentary (Deban and Wake 2000a; Lorenz Elwood and Cundall 1994), inserting on the anterior edge of the first hypohyal (Lorenz Elwood and Cundall 1994).

Hyobranchial Retractors muscles group description:

- *Muscle rectus cervicis* (MRC) is divided into two portions: the *superficialis* (MRCsup) (Fig. 2, B4) and the *profundus* (MRCpro) (Fig. 2, B5) (Deban and Wake 2000a). The superficial portion originates on the sternum and inserts on the first ceratobranchials (Deban and Wake 2000a; Krogh and Tanner 1972;). The deep portion originates dorsally on the hyobranchial bones and inserts on the *rectus abdominis* (Schwarz et al. 2020a; Larsen and Guthrie 1975). This muscle is very long, often composed of different sections of fibers.

Supplementary Fig. S1 provides a visualization of the different functional groups.

After dissection, muscles were weighed using a digital scale (Mettler Toledo, Balance XS105, precision 0.01 mg). Next, fiber lengths were obtained by submerging the muscles in a 30% nitric acid solution. After 24 hours, the connective tissue is digested, and the fibers become easily detachable. The muscle fibers were then transferred to a 50% glycerol solution to stop the digestion and preserve the fibers. Photos of the fibers were taken using a binocular microscope (Leica MZ16) with a camera (JENOPTIK GRYPHAX), and the fiber lengths were quantified using IMAGEJ 1.54j software (Wayne Rasband, National Institutes of Health, Bethesda, MD, USA). For each muscle, the length of ten haphazardly selected fibers was measured, and the average fiber length for each muscle was calculated.

Finally, the physiological cross-sectional area (PCSA) of each muscle was calculated as follows:


\begin{eqnarray*}
\textit{PCSA}\,\left( {c{{m}^2}} \right) = \frac{{\textit{mass}\left( g \right)/muscular\,\textit{density}\left( {\frac{g}{{c{{m}^3}}}} \right)}}{{\textit{fiber}\,\textit{length}\left( {cm} \right)}}
\end{eqnarray*}

With a muscular density of 1.06 g.cm^-3^ (Mendez and Keys 1960).

### Statistical analysis

To investigate the effects of differences induced by different developmental strategies on the myology of the feeding system, we proceeded as follows: first, muscles were grouped into functional categories (as outlined earlier). Life cycles were biologically categorized as in Fig. 1: ecological transition (yes or no), morphological transformation (yes or no), and adult habitat (terrestrial, aquatic, semi-aquatic).

In this study, the comparisons do not primarily concern species as whole entities but rather individuals or groups of individuals representing distinct developmental strategies. Individuals from the same species may belong to different developmental categories (e.g., paedomorphic vs. metamorphic forms) and were therefore treated as separate biological entries. Conversely, when multiple individuals from the same species fell into the same developmental category, their measurements were averaged to generate a single representative value for that category.

All subsequent analyses were conducted in R (R Core Team v4.5.2) and as a proxy for size, the variable “Head length” (HL) was used as a covariate. All muscular and morphological variables were log10-transformed before analysis to meet the assumptions of normality and homoscedasticity (See Supplementary Table S4).

#### Phylogeny

Since species data are not independent, phylogeny must be considered in the analyses (Felsenstein 1985). To address this, we used the time-calibrated phylogeny published by Stewart and Wiens (2025). This phylogeny was constructed using a combined phylogenomic and supermatrix approach. The tree topology was inferred using maximum likelihood method, and divergence times were estimated using penalized likelihood implemented in treePL (Smith and O’Meara 2012). A polytomy was created for the species with two life cycles in our sample (*Ambystoma mexicanum* and *Ambystoma andersoni*).

#### Fit the model of evolutionary processes to the data

We tested the effects of three biological factors, morphological transformation, ecological transition, and adult habitat on the volume of each muscle group and on the PCSA of each group respectively. Head length (HL) was used as a covariable. We first fit the model of evolutionary processes to the data using *mvMORPH* package (v1.2.1) (Clavel et al. 2015). We compared the Generalized Information Criterion (GIC) (Konishi and Kitagawa 1996) across four different models (Ornstein-Uhlenbeck, Brownian motion, Early Burst, and Pagel’s lambda transformation) and selected the model with the lowest GIC criterion. The “mvgls” function of the *mvMORPH* package was used to conduct this analysis (Clavel et al. 2015). The chosen model was used for the remaining analyses.

#### Phylogenetic principal component analyses

To visualize the distribution of individuals or species within myological space, identify major patterns in muscle morphology, and evaluate potential relationships between muscle structure morphological transformation, ecological transition and adult habitat, phylogenetic principal component analyses (PCA) were conducted using both muscle volume and PCSA data separately. To remove size effects and make differences comparable among groups, the phylogenetic PCAs were performed on the phylogenetic regression of scaled muscle variables on scaled head length. This was done using the “mvgls.pca” function from the *mvMORPH* package (Clavel et al. 2015).

#### Phylogenetic multivariate analysis of covariance

To test how morphological transformation, ecological transition, adult habitat, and HL influence muscular traits (volume and PCSA separately) while accounting for the shared evolutionary history of species, we conducted a phylogenetic multivariate analysis of covariance (MANCOVA). In this analysis, morphological transformation, ecological transition, and adult habitat were treated as independent variables, HL as a covariate and the different muscular traits as dependent variables. To incorporate phylogenetic relationships into the analysis, we used the “manova.gls” function from the *mvMORPH* package (Clavel et al. 2015) and the effect size of each variable was estimated using Wilks’ τ² statistic, computed with the “effectsize” function from the same package. The MANCOVA analyses were conducted using Wilks’ Lambda as the test statistic with 999 permutations and a Type II approach. By doing so, we aimed to assess whether differences in muscle properties (volume and PCSA, respectively) are significantly associated with evolutionary shifts in life-cycle strategies.

#### Phylogenetic analysis of covariance

Finally, we performed phylogenetic analysis of covariance (ANCOVA) to test the effects of morphological transformation, ecological transition, adult habitat, and HL on muscle volume and PCSA separately. Generalized least squares (GLS) models were fitted using the “gls” function from the *nlme* package (v3.1.168) (Pinheiro and Bates 2000, Pinheiro and Bates 2024) incorporating Martins and Hansen’s (1997) phylogenetic correlation structure via the corMartins correlation structure to account for phylogenetic signal in the residuals. Type II ANOVA tables were extracted for each muscle group using the “Anova” function from the *car* package (v3.1.3) (Fox and Weisberg 2019). For traits that showed a significant ecological effect in the ANCOVA, we performed post-hoc pairwise comparisons. Post-hoc tests were implemented using estimated marginal means and pairwise contrasts between ecological categories were computed using Tukey tests.

### AI used in the study

Portions of the code were developed with the assistance of ChatGPT (GPT-4 model, free version available in 2025; OpenAI).

## Results

### Comparative analysis of hyobranchial system musculature

Some differences were observed between paedomorphic specimens and those that undergo complete metamorphosis. In their paedomorphic form, species such as *Ambystoma andersoni, Ambystoma mexicanum, Proteus anguinus, Siren intermedia*, and *Siren lacertina*, the *quadratopectoralis* muscle is absent. Instead, these species possess a *branchiohyoideus* muscle, which links the hyoid and branchial systems, resulting in an integrated hyobranchial system. Moreover, in paedomorphic forms, the *subhyoideus* muscle was not found during dissections. Interestingly, a *pterygoideus* muscle (Carroll and Holmes 1980) was identified in *Siren* species, adding to the distinctive muscular organization observed in these paedomorphic forms.

### Model fit

The model retained for all muscle volume and PCSA analysis was Ornstein-Uhlenbeck (Uhlenbeck and Ornstein 1930), with the lowest GIC estimated using maximum likelihood (Table 1 and Supplementary Table 5 for details).

**Table 1:** The table presents the estimated characteristics of the Volume and PCSA models (Ornstein-Uhlenbeck), along with GIC and Log-likelihood values, for three factors: ecological transition, morphological transformation, and adult habitat.

	Volume		PCSA	
	GIC	Log-likelihood	GIC	Log-likelihood
**Ecological transition**	8.9	21.1	19.3	15.4
**Morphological transformation**	11.2	20.1	20.7	15.0
**Adult habitat**	4.4	24.9	8.4	23.1

### Phylogenetic principal component analysis

**Fig. 3:**
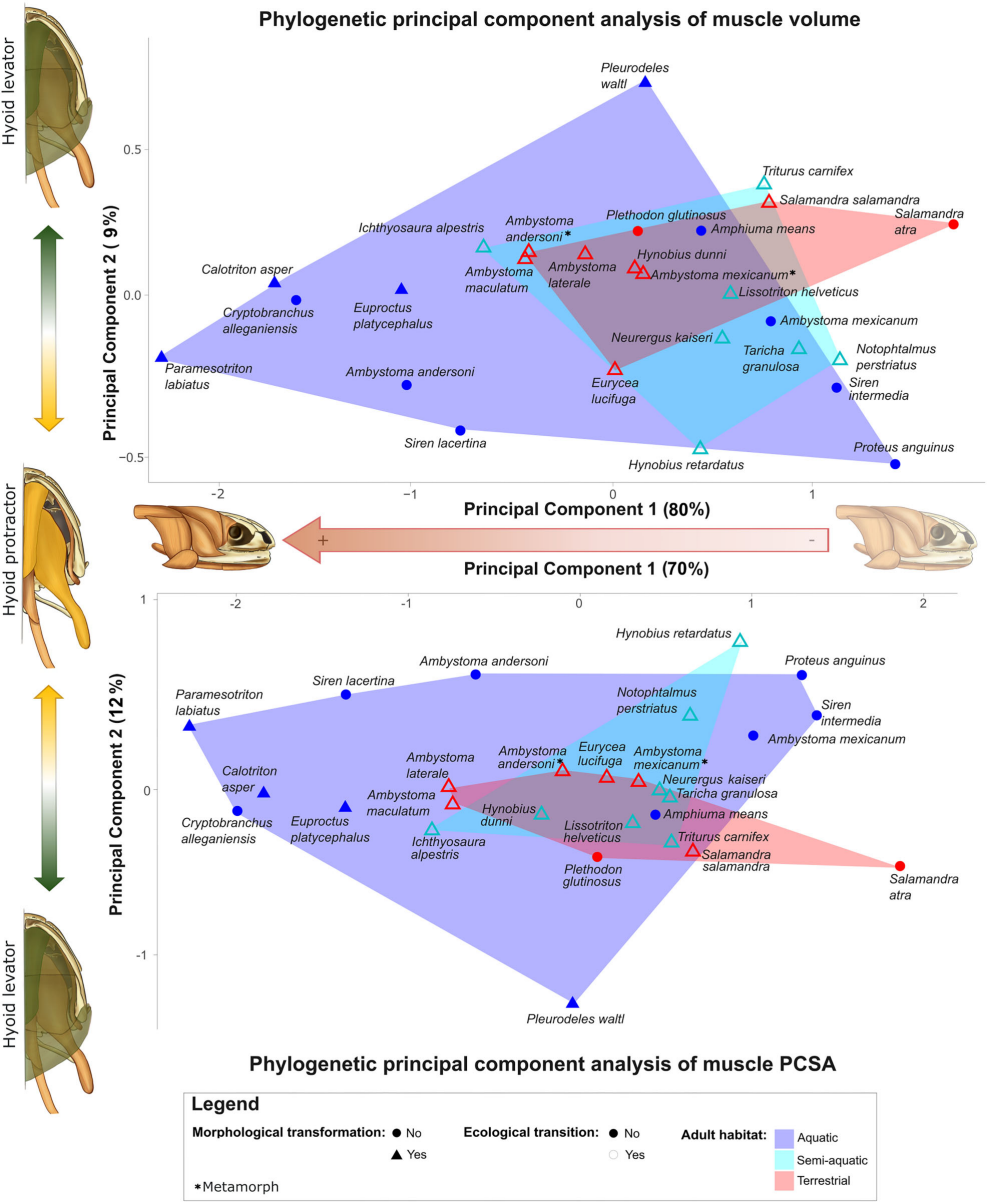
Phylomorphospace of PCA for muscle characteristics. Top: muscle volume; bottom: muscle PCSA. Each point represents an individual. Filled points indicate individuals without an ecological transition, while empty points indicate individuals where a transition occurred. Point shape denotes morphological transformation (circles: No; triangles: Yes). Colored polygons indicate adult habitat type (dark blue: Aquatic, light blue: Semi-aquatic, red: Terrestrial). Axes show principal components (PC1, PC2) and their explained variance. Silhouettes provide a schematic interpretation of the principal component axes. PC1 represents a shared direction of variation in muscle volume and PCSA, while PC2 represents opposing patterns of variation between two muscle groups observed in both analyses.

The first two principal components (PC) of the phylogenetic PCA, performed on both muscle volume and PCSA values separately (Fig. 3) accounted for 89% of the overall variance for the volume and 82% of the overall variance for PCSA measurements. For the volume, the first two axes account for 80% and 9% of the overall variance respectively. For the PCSA, the first two axes account for 70% and 12% of the overall variance respectively (Table 2).

**Table 2:** Phylogenetic principal component analysis results for muscle volume and PCSA corrected by head length.

		PC1	PC2	PC3
**Muscle volume**	**eigenvalue**	0.0058	0.0006	0.0004
	**% of variance**	80	9	5
	Vol_HyoLev	-0.36	0.57	0.64
	Vol_HyoPro	-0.44	-0.65	0.51
	Vol_HyoRet	-0.50	-0.33	-0.36
	Vol_MouthOp	-0.44	0.23	-0.39
	Vol_MouthClo	-0.49	0.30	-0.22
**Muscle PCSA**	**eigenvalue**	0.0069	0.0011	0.0010
	**% of variance**	70	12	11
	PCSA_HyoLev	-0.34	-0.50	0.74
	PCSA_HyoPro	-0.41	0.68	0.43
	PCSA_HyoRet	-0.50	0.34	-0.29
	PCSA_MouthOp	-0.48	-0.29	-0.24
	PCSA_MouthClo	-0.48	-0.30	-0.35

Eigenvalues, percentage of explained variance, and loadings of individual muscles on the first three principal components are shown (results modified after scaling the variables before the PCA).

For both muscle volume and PCSA, the first principal component reflects muscle robsutness, with all muscles loading in the same direction. This axis separates aquatic individuals from terrestrial and semi–aquatic ones. Aquatic individuals cluster towards the negative side of PC1, with the exception of some paedomorphic species (*Proteus anguinus, Siren intermedia*, and *Ambystoma mexicanum*). After correcting for head size, this pattern suggests that aquatic species generally possess relatively larger muscle volumes and higher force–generating capacity compared to terrestrial and semi–aquatic taxa, which are positioned toward the positive side of PC1 and exhibit comparatively smaller muscles.

Aquatic species also show greater overall variation, and the second principal component tends to separate paedomorphic aquatic species, characterized by relatively larger hyoid protractors and smaller hyoid elevators, from other aquatic taxa (Fig. 3). This axis reflects differences in the relative contribution of individual muscle groups rather than overall muscle size. In particular, it contrasts hyoid elevators and hyoid protractors, which load in opposite direction, indicating variation in the functional organization of the hyoid apparatus.

### Phylogenetic multivariate analysis of covariance

The multivariate analysis of covariance revealed contrasting effects of morphological transformation, ecological transition, the adult habitat and the head length on muscle architecture (Table 3).

**Table 3:** Results of MANCOVAs testing the effects of ecological transition (Eco), morphological transformation (Morpho), and adult habitat (Habitat) on muscle volume and PCSA, with head length (HL) as a covariate.

	Muscle volume						Muscle PCSA					
	Wilk’s Lambda	F-Value	*P*-value	Num Df	Den DF	Effectsize	Wilk’s Lambda	F-Value	*P*-value	Num Df	Den DF	Effectsize
	**Ecological transition**											
**Eco**	0.45	4.65	**0.006**	5	19	0.55	0.60	2.58	0.061	5	19	0.40
**HL**	0.19	16.60	**0.001**	5	19	0.81	0.24	11.94	**0.001**	5	19	0.76
Eco*HL	0.47	4.35	**0.008**	5	19	0.53	0.53	3.31	**0.025**	5	19	0.47
	**Morphological transformation**											
**Morpho**	0.38	6.17	**0.001**	5	19	0.62	0.52	3.46	**0.022**	5	19	0.47
**HL**	0.16	19.70	**0.001**	5	19	0.84	0.23	13.00	**0.001**	5	19	0.77
Morpho*HL	0.69	1.67	0.191	5	19	0.31	0.67	1.89	0.144	5	19	0.33
	**Adult habitat**											
**Habitat**	0.23	3.75	**0.002**	10	34	0.52	0.29	2.89	**0.010**	10	34	0.46
**HL**	0.23	11.65	**0.001**	5	17	0.77	0.28	8.67	**0.001**	5	17	0.72
Habitat*HL	0.45	1.65	0.133	10	34	0.33	0.51	1.36	0.239	10	34	0.28

Wilks' Lambda, F-values, P-values, degrees of freedom (Num DF, Den DF) and effect size are reported for main effects and interactions. Significant results are shown in bold.

For muscle volume, head length (HL) has a very strong effect (*P* < 0.001), and within each model it shows a larger effect size than any other factor. Ecological transition also has a significant effect (Wilk’s λ = 0.45, F = 4.65, *P* = 0.006), as does morphological transition (Wilk’s λ = 0.38, F = 6.17, *P* = 0.001) and adult habitat (Wilk’s λ = 0.23, F = 3.75, *P* = 0.002). The interaction between ecological transition and HL is significant as well (*P* = 0.008), showing that the relationship between HL and muscle volume differs between individuals undergoing an ecological transition and those that are not. In contrast, the interactions between HL and morphological transition or adult habitat are not significant.

Patterns are similar for PCSA. HL is significant across all (*P* < 0.001), and its effect size is although larger than for other factors. Morphological transition (Wilk’s λ = 0.52, F = 3.46, *P* = 0.022) and adult habitat (Wilk’s λ = 0.29, F = 2.89, *P* = 0.010) both show significant effects on PCSA, while the effect of ecological transition is weaker (Wilk’s λ = 0.60, F = 2.58, *P* = 0.061). The interaction between ecological transition and HL is significant as well (*P* = 0.025).

### Phylogenetic analysis of covariance

The ANCOVA results (Table 4) show that head length (HL) has a strong significant effect on every muscle group, for both muscle volume and PCSA (all *P* < 0.001).

**Table 4:** Results of ANCOVA testing the effects of ecological transition (Eco), morphological transformation (Morpho), and adult habitat (Habitat) on muscle volume and PCSA, with head length (HL) as a covariate.

			Muscle volume		Muscle PCSA	
		Df	Chisq	Pr(>Chisq)	Chisq	Pr(>Chisq)
		**Ecological transition**				
**Hyoid levator**	**Eco**	1	0.00	0.944	0.23	0.632
	**HL**	1	81.63	**0.001**	60.45	**0.001**
**Hyoid protractor**	**Eco**	1	0.88	0.348	0.00	0.970
	**HL**	1	56.80	**0.001**	46.60	**0.001**
**Hyoid retractor**	**Eco**	1	2.14	0.143	2.48	0.115
	**HL**	1	49.20	**0.001**	37.56	**0.001**
**Mouth opening**	**Eco**	1	1.37	0.242	1.00	0.316
	**HL**	1	72.27	**0.001**	44.25	**0.001**
**Mouth closing**	**Eco**	1	2.85	0.092	2.32	0.128
	**HL**	1	52.69	**0.001**	43.56	**0.001**
		**Morphological transformation**				
**Hyoid levator**	**Morpho**	1	3.86	**0.049**	2.78	0.095
	**HL**	1	101.33	**0.001**	68.19	**0.001**
**Hyoid protractor**	**Morpho**	1	0.31	0.578	0.13	0.718
	**HL**	1	56.60	**0.001**	42.75	**0.001**
**Hyoid retractor**	**Morpho**	1	0.00	0.966	0.19	0.666
	**HL**	1	45.59	**0.001**	38.36	**0.001**
**Mouth opening**	**Morpho**	1	0.84	0.359	0.89	0.347
	**HL**	1	75.46	**0.001**	48.97	**0.001**
**Mouth closing**	**Morpho**	1	0.72	0.397	0.92	0.337
	**HL**	1	55.92	**0.001**	48.79	**0.001**
		**Adult habitat**				
**Hyoid levator**	**Habitat**	2	2.49	0.288	1.32	0.516
	**HL**	1	65.40	**0.001**	17.25	**0.001**
**Hyoid protractor**	**Habitat**	2	3.33	0.190	0.50	0.777
	**HL**	1	45.60	**0.001**	16.24	**0.001**
**Hyoid retractor**	**Habitat**	2	7.09	**0.029**	0.39	0.821
	**HL**	1	41.86	**0.001**	12.26	**0.001**
**Mouth opening**	**Habitat**	2	5.73	0.057	4.20	0.123
	**HL**	1	60.30	**0.001**	14.95	**0.001**
**Mouth closing**	**Habitat**	2	6.39	**0.041**	2.73	0.255
	**HL**	1	41.85	**0.001**	16.11	**0.001**

Degrees of freedom (Df), Chi-square values, and associated P-values are reported. Significant results are shown in bold.

**Table 5.** Post-hoc pairwise comparisons among ecological categories (Aquatic, Semi-Aquatic, Terrestrial) for traits showing significant ANCOVA results.

	Contrast	Estimate	t-ratio	*P*-value
**Volume Hyoid retractor**	Aquatic -Semi-Aquatic	0.465	2.254	0.08
	Aquatic-Terrestrial	0.388	2.262	0.08
	Semi-Aquatic-Terrestrial	-0.077	-0.389	0.92
**Volume Mouth opening**	Aquatic -Semi-Aquatic	0.351	2.251	0.08
	Aquatic-Terrestrial	0.224	1.721	0.22
	Semi-Aquatic-Terrestrial	-0.128	-0.834	0.69
**Volume Mouth Closing**	Aquatic -Semi-Aquatic	0.431	2.494	0.05
	Aquatic-Terrestrial	0.211	1.468	0.33
	Semi-Aquatic-Terrestrial	-0.220	-1.297	0.41

For each significant ANCOVA, post-hoc contrasts are reported with their estimated difference, t-ratio, and associated *P*-value.

In contrast, ecological transition does not have a significant effect on any individual muscle, whether considering volume or PCSA (all *P* > 0.05). Morphological transformation shows a marginal effect only for the hyoid levator in terms of muscle volume (χ² = 3.86, *P* = 0.049), but no other muscle group is affected. For PCSA, this effect becomes non–significant (*P* = 0.095). Adult habitat also does not significantly explain variation in any muscle when PCSA is considered (all *P* > 0.05). For muscle volume, adult habitat shows weak effects on the hyoid retractor (χ² = 7.09, *P* = 0.029) and mouth–closing muscles (χ² = 6.39, *P* = 0.041), but these effects are small compared to the influence of HL.

Post-hoc pairwise comparisons (Table 5) were performed for the three muscle groups for which ANCOVA returned significant or marginal results. These revealed no significant differences between ecological categories. The hyoid retractor showed marginal differences tending to differentiate aquatic taxa from other ones with different types of habitats (both *P* = 0.08). For the mouth-opening and mouth-closing muscles, the only contrasts approaching significance tend to differentiate semi-aquatic taxa from aquatic ones (*P* = 0.08 and *P* = 0.05, respectively), while all other comparisons being non-significant.

## Discussion

Life-cycle variation (ecological transition and morphological transformation), body size, and adult habitat are well-known to drive variation in the morphology of skull bony structures in Caudata (Fabre et al. 2020; Louppe et al. 2025). Our results show that similar factors also affect the feeding musculature. Across all statistical models, head length (HL) emerged as the strongest and most consistent predictor of both muscle volume and PCSA, indicating a strong correlation between skull size and feeding muscle architecture. This relationship likely reflects spatial constraints associated with muscle attachment. However, it would be interesting to further investigate the relationships between size, muscle properties and functional performance in the future.

Our first hypothesis predicted that salamanders that shift from aquatic to terrestrial environments would show differences in feeding muscle architecture compared with species that remain in a single environment. As predicted, the results of the phylogenetic MANCOVA revealed a significant effect of ecological transition on overall muscle architecture. This result indicates that experiencing an ecological transition is associated with a reorganization of the feeding system (Schwarz et al. 2020b; Lauder and Reilly 1990; Ziermann and Diogo 2013). Species that transition between environments must perform feeding under two distinct sets of constraints, which may create a functional trade-off compared with species restricted to a single medium (Heiss, Aerts and Van Wassenbergh 2018). Moreover, the significant interaction between head length and ecological transition suggests that the relationship between head length and muscle properties is context dependent. However, the present results do not allow inference of the underlying functional mechanisms driving this pattern. It is interesting to note that similar context-dependent relationships between morphology and ecological conditions have been reported in salamanders and other amphibians, where environmental transitions can alter scaling relationships between cranial traits and feeding-related structures (Heiss, Aerts and Van Wassenbergh 2018; Laudet 2011; Ptatscheck et al. 2025). This suggests that the same head size can be associated with different muscle configurations depending on environmental context, likely due to additional developmental constraints, but this hypothesis requires further testing.

Our second hypothesis hypothesized that species undergoing a complete post-hatching metamorphosis (biphasic) would differ in muscle architecture from species that do not undergo a complete metamorphosis, either because they develop directly without a larval stage (direct developers) or because they retain larval features into adulthood (paedomorphic species). This can be appreciated through the results of the phylogenetic PCA, where PC2 separates aquatic species, with paedomorphic taxa differing from other aquatic species encountering a complete metamorphosis. This axis reflects differences in the functional organization of muscle groups, with paedomorphic species showing a greater emphasis on hyoid protractor muscles, whereas non-paedomorphic aquatic salamanders rely more on hyoid elevator muscles. These results are in line with the anatomical differences observed during dissections, where we found that paedomorphic species lack the quadratopectoralis muscle, which is part of the hyoid elevator group in non-paedomorphic species. Instead, they possess a branchiohyoideus muscle, linked to the branchial system, and belonging to the hyoid protractor group. These muscle configurations reflect the retention of larval traits related to gill bearing and hyobranchial function. Because paedomorphic species retain external gills (Bonett and Ledbetter 2022), the hyoid musculature plays roles in both feeding and branchial respiration (Lauder and Bradley Shaffer 1985). These dual functional demands may constrain muscle architecture and explain why paedomorphic species differ from other aquatic salamanders, even though their adult habitats are similar. Overall, these results show that metamorphosis seems to be a developmental constraint shaping feeding muscle organization.

Finaly our third hypothesis predicted that adult habitat influences feeding muscle architecture. We expected terrestrial species to exhibit stronger jaw adductors for prey capture on land (Deban and Wake 2000b), aquatic species to retain suction-feeding adaptations with a more developed hyoid apparatus (Deban and Wake 2000a), and semi-aquatic species to show intermediate conditions. The results of both the phylogenetic PCA and MANCOVA confirmed a significant effect of habitat on overall muscle volume and PCSA. Aquatic adults generally exhibit larger and more powerful feeding muscles, consistent with the increased mechanical demands of suction feeding in water, where fluid density and viscosity impose strong hydrodynamic constraints (Heiss et al. 2013). The feeding mechanism relies on hyobranchial depression, where downward movement of the hyoid apparatus generates negative pressure that draws prey into the buccal cavity (Deban and Wake 2000a). Its efficiency depends on the coordinated action of hyoid musculature and the skeletal elements of the hyoid, which together form a lever system optimizing depression movements (Deban and Wake 2000a; Lauder and Bradley Shaffer 1985; Lauder and Reilly 1990; Deban et al. 2001). At the level of individual muscles, phylogenetic ANCOVAs revealed only weak effects, mainly associated with the hyoid retractors and jaw adductor muscles. This may be explained by the physical properties of the aquatic environment, which is more viscous and therefore requires greater force for rapid mouth closure and effective hyoid leverage during prey capture.

Statistical tests at the multivariate level show that ecological transition, morphological transformation, and adult habitat all significantly influence the overall feeding muscle architecture, indicating that life-history strategies shape the global organization of cranial feeding muscles. In contrast, when examining individual muscles while taking into account head length, these ecological and developmental factors rarely show differences, indicating that size remains the dominant predictor. This suggests that ecological effects are not localized to specific muscles but instead distributed across the entire muscular system. While ecological signals are strong enough to coordinate variation among muscles at the system level, they are too diffuse to generate clear, muscle-specific differences This pattern is consistent with the high muscular plasticity and functional versatility of salamander feeding systems, which reflects the capacity of cranial muscles to adjust their relative development, activation patterns, and functional roles depending on ecological context. This includes reversible shifts between suction feeding and tongue-based feeding in species that seasonally transition between habitats (Heiss et al. 2016).

Several limitations should be considered when interpreting these results. First, the sample size may reduce statistical power, particularly for detecting subtle differences among ecological categories. Second, destructive dissections limit access to specimens, especially rare or threatened taxa, whereas skeletal data can increasingly be obtained non-destructively using micro-CT scanning. Future work should therefore aim to further investigate the relationships between size, muscle properties, and functional performance. Integrating our muscle dataset with three-dimensional cranial morphometric data would enable the investigation of potential coevolutionary patterns between the muscular system and skull skeletal elements (Fabre et al. 2020; Louppe et al. 2025).

Additionally, seasonal plasticity and intraspecific variation (e.g., sexual dimorphism and facultative paedomorphosis) may influence muscle architecture. Given the constraints in specimen availability and incomplete metadata regarding sex, we were unable to explicitly test for sexual dimorphism. While sexual dimorphism in cranial morphology has been reported in some salamander species (Alarcón-Ríos et al. 2017; Wang et al. 2023), its magnitude is species-dependent and generally assumed to be modest relative to interspecific variation. Its potential influence on our results, however, cannot be excluded. Complete sampling across sexes and across aquatic and terrestrial phases could further help elucidate the extent of reversible plasticity in musculature.

To conclude, this study demonstrates that head size is an important determinant of feeding muscle morphology in salamanders. Life-cycle strategies (ecological transition and morphological transformation) and adult habitat influence the global configuration of the musculature but do not generate strong, isolated differences in individual muscles groups. Functional ecology, particularly the demands of suction feeding, appears to drive muscular organization, while constraints may limit divergence at finer anatomical scales. Understanding the evolution of salamander feeding systems will require integrating multiple levels of variation, from within-individual plasticity to macroevolutionary transitions, and combining skeletal and muscular perspectives to capture the full complexity of their ecological and functional diversity.

## Supplementary Material

icag040_Supplemental_File

## Data Availability

The data and script used for statistical analysis is publicly available in the GitHub repository: https://github.com/MorganeTlds/Impact-of-life-cycle-variation-on-feeding-system-musculature-in-Caudata.git

## Notes

From the symposium “The evolutionary consequences of life cycle complexity” presented at the annual meeting of the Society for Integrative and Comparative Biology, January 3–7th, 2026.
