# Supplementary material for: Impact of Life-Cycle Variation on Feeding System Musculature in Caudata
Source: Integr Comp Biol. 2026 May 7;66:icag040. doi: 10.1093/icb/icag040 (PMC13196598; doi:10.1093/icb/icag040)
Supplement: icag040_Supplemental_File [file icag040_supplemental_file.docx]

**Supplementary information**

Supplementary Table 1: Anatomical literature classified by species studied in this article, and at which developmental stage.

| Family | Species | Stage | Reference |
| --- | --- | --- | --- |
| Ambystomatidae | *Ambystoma annulatum* | Adult | (Krogh & Tanner, 1972) |
| Ambystomatidae | *Ambystoma cingulatum* | Adult | (Krogh & Tanner, 1972) |
| Ambystomatidae | *Ambystoma dumerilii* | Adult | (Lauder & Bradley Shaffer, 1985) |
| Ambystomatidae | *Ambystoma gracile* | Adult | (Krogh & Tanner, 1972) |
| Ambystomatidae | *Ambystoma jeffersonianum* | Adult | (Krogh & Tanner, 1972) |
| Ambystomatidae | *Ambystoma mabeei* | Adult | (Krogh & Tanner, 1972) |
| Ambystomatidae | *Ambystoma macrodactylum* | Adult | (Krogh & Tanner, 1972) |
| Ambystomatidae | *Ambystoma maculatum* | Adult | (Carroll & Holmes, 1980) |
| Ambystomatidae | *Ambystoma maculatum* | Adult | (Krogh & Tanner, 1972) |
| Ambystomatidae | *Ambystoma mexicanum* | Adult | (Lauder & Bradley Shaffer, 1985) |
| Ambystomatidae | *Ambystoma mexicanum* | Adult | (Ziermann & Diogo, 2013) |
| Ambystomatidae | *Ambystoma mexicanum* | Larvae and embryo | (Ziermann & Diogo, 2013) |
| Ambystomatidae | *Ambystoma opaeum* | Adult | (Krogh & Tanner, 1972) |
| Ambystomatidae | *Ambystoma ordinarium* | Adult | (Lauder & Bradley Shaffer, 1985) |
| Ambystomatidae | *Ambystoma rosaceum* | Adult | (Krogh & Tanner, 1972) |
| Ambystomatidae | *Ambystoma talpoideum* | Adult | (Krogh & Tanner, 1972) |
| Ambystomatidae | *Ambystoma texanum* | Adult | (Krogh & Tanner, 1972) |
| Ambystomatidae | *Ambystoma tigrinum* | Adult | (Krogh & Tanner, 1972) |
| Ambystomatidae | *Ambystoma tigrinum* | Adult | (Lauder & Bradley Shaffer, 1985) |
| Ambystomatidae | *Ambystoma tigrinum melanosticum* | Adult | (Krogh & Tanner, 1972) |
| Ambystomatidae | *Ambystoma tigrinum melanosticum* | Adult | (Larsen & Guthrie, 1975) |
| Ambystomatidae | *Ambystoma tigrinum nebulosum* | Adult | (Krogh & Tanner, 1972) |
| Ambystomatidae | *Ambystoma tigrinum tigrinum* | Adult | (Krogh & Tanner, 1972) |
| Ambystomatidae | *Dicamptoon ensatus* | Adult | (Krogh & Tanner, 1972) |
| Amphiumidae | *Amphiuma tridactylum* | Adult | (Carroll & Holmes, 1980) |
| Amphiumidae | *Amphiuma tridactylum* | Adult | (Erdman & Cundall, 1984) |
| Cryptobranchidae | *Andrias japonicus* | Adult | (Matsumoto, Fujiwara, & Evans, 2024) |
| Cryptobranchidae | *Cryptobranchus alleganiensis* | Adult | (Lorenz Elwood & Cundall, 1994) |
| Cryptobranchidae | *Cryptobranchus alleganiensis* | Adult | (Carroll & Holmes, 1980) |
| Hynobiidae | *Hynobius naevius* | Adult | (Carroll & Holmes, 1980) |
| Hynobiidae | *Hynobius retardatus* | Adult | (Carroll & Holmes, 1980) |
| Plethodontidae | *Desmogmathus monticola* | Adult | (Hinderstein, 1971) |
| Plethodontidae | *Desmognathus aeneus* | Adult | (Hinderstein, 1971) |
| Plethodontidae | *Desmognathus fuscus* | Adult | (Hinderstein, 1971) |
| Plethodontidae | *Desmognathus ochrophaeus* | Adult | (Hinderstein, 1971) |
| Plethodontidae | *Desmognathus quadramaculatus* | Adult | (Deban & Richardson, 2017) |
| Plethodontidae | *Desmognathus quadramaculatus* | Adult | (Hinderstein, 1971) |
| Plethodontidae | *Desmognathus wrighti* | Adult | (Hinderstein, 1971) |
| Plethodontidae | *Hydromantes italicus* | Adult | (Bauer, 1997) |
| Plethodontidae | *Leurognathus marmoratus* | Adult | (Hinderstein, 1971) |
| Plethodontidae | *Phaeognathus hubrichti* | Adult | (Hinderstein, 1971) |
| Proteidae | *Necturus maculosus* | Adult | (Bauer, 1997) |
| Proteidae | *Necturus maculosus* | Adult | (Carroll & Holmes, 1980) |
| Rhyacotritonidae | *Rhyacotriton olympicus* | Adult | (Krogh & Tanner, 1972) |
| Salamandridae | *Euproctus asper* | Adult | (Bauer, 1997) |
| Salamandridae | *Ichtyosora alpestris* | Adult | (Heiss, Handschuh, Aerts, & Van Wassenbergh, 2016) |
| Salamandridae | *Lissotriton vulgaris* | Adult | (Heiss, Handschuh, Aerts, & Van Wassenbergh, 2016) |
| Salamandridae | *Notophtamus viridescens* | Adult | (Bauer, 1997) |
| Salamandridae | *Notophtamus viridescens* | Adult | (Carroll & Holmes, 1980) |
| Salamandridae | *Salamandra salamandra* | Adult | (Bauer, 1997) |
| Salamandridae | *Salamandra salamandra* | Adult | (Krogh & Tanner, 1972) |
| Salamandridae | *Salamandra salamandra* | Larvae | (Bauer, 1997) |
| Salamandridae | *Taricha granulosa* | Adult | (Bauer, 1997) |
| Salamandridae | *Taricha torosa* | Adult | (Bauer, 1997) |
| Salamandridae | *Triturus cristatus* | Adult | (Bauer, 1997) |
| Salamandridae | *Triturus cristatus* | Larvae | (Bauer, 1997) |
| Salamandridae | *Triturus helveticus* | Adult | (Bauer, 1997) |
| Salamandridae | *Triturus karelini* | Adult | (Bauer, 1997) |
| Salamandridae | *Triturus marmoratus* | Adult | (Bauer, 1997) |
| Salamandridae | *Triturus vulgaris* | Adult | (Bauer, 1997) |
| Salamandridae | *Triturus vulgaris* | Larvae | (Bauer, 1997) |
| Sirenidae | *Siren intermedia* | Adult | (Schwarz, Konow, Tolosa Roba, & Heiss, 2020) |
| Sirenidae | *Siren lacertina* | Adult | (Carroll & Holmes, 1980) |

| **Id** | **Species** | **Sex** | **SVL** | **HL** | **Collection** |
| --- | --- | --- | --- | --- | --- |
|  | *Ambystoma_andersoni* | F | 82.75 | 19.4 | AH&MM personal collection |
|  | *Ambystoma_andersoni_metamorph* | - | 84.63 | 16.26 | AH&MM personal collection |
| US013 | *Ambystoma_laterale* | F | 38.855 | 8.06 | AH&MM personal collection |
| US026 | *Ambystoma_maculatum* | M | 80.9 | 16.265 | AH&MM personal collection |
|  | *Ambystoma_mexicanum* | F | 107.85 | 30.405 | AH&MM personal collection |
| US025 | *Ambystoma_mexicanum_metamorph* | M | 105.975 | 25.12 | AH&MM personal collection |
| Paris | *Ambystoma_mexicanum_metamorph* | - | 104 | 21.05 | AH&MM personal collection |
|  | *Amphiuma_means* | F | 275 | 23.605 | AH&MM personal collection |
|  | *Calotriton_asper* | - | 74.41 | 12.47 | AH&MM personal collection |
| US027 | *Cryptobranchus_alleganiensis* | - | 90.52 | 15.095 | AH&MM personal collection |
|  | *Euproctus_platycephalus* | - | 40.48 | 8.84 | AH&MM personal collection |
| US006 | *Eurycea_lucifuga* | F | 61.05 | 10.545 | AH&MM personal collection |
|  | *Hynobius_dunni* | - | 64 | 14.9 | AH&MM personal collection |
| M1 | *Hynobius_retardatus* | M | 43.305 | 9.875 | AH&MM personal collection |
| 1016783 | *Ichthyosaura_alpestris* | M | 42.62 | 8.4 | AH&MM personal collection |
| 4 | *Lissotriton_helveticus* | F | 34.945 | 8.27 | AH&MM personal collection |
| US009 | *Neurergus_kaiseri* | - | 42.57 | 10.245 | AH&MM personal collection |
| US012 | *Notophthalmus_perstriatus* | M | 33.605 | 9.59 | AH&MM personal collection |
|  | *Paramesotriton_labiatus* | - | 67.56 | 11.6 | AH&MM personal collection |
| US002 | *Plethodon_glutinosus* | F | 63.93 | 12.49 | AH&MM personal collection |
|  | *Pleurodeles_waltl* | F | 66.77 | 14.72 | AH&MM personal collection |
|  | *Proteus_anguinus* | F | 129.51 | 20.33 | AH&MM personal collection |
| 1016692 | *Salamandra_atra* | M | 55.235 | 13.92 | NMBE collection |
| M2 | *Salamandra_salamandra* | F | 89.78 | 24.25 | AH&MM personal collection |
| Bernardezi | *Salamandra_salamandra_bernardezi* | - | 62.23 | 12.78 | AH&MM personal collection |
|  | *Siren_intermedia* | - | 40.06 | 9.305 | AH&MM personal collection |
| SIR2 | *Siren_lacertina* | - | 292.37 | 29.71 | AH&MM personal collection |
| US003 | *Taricha_granulosa* | - | 42.92 | 10.83 | AH&MM personal collection |
| SHIK234 | *Triturus_carnifex* | F | 56.355 | 11.19 | AH&MM personal collection |

Supplementary Table 2: Detailed list of the specimens dissected in this study. F: Female; M: Male; AH & MM: Anthony Herrel and Mark Mandica, NMBE: Naturhistorisches Museum Bern

Supplementary Table 3: Muscles associated with the feeding system and their synonyms.

| ***Muscle*** | ***Division*** | ***Abbreviation*** | ***Synonyms*** |
| --- | --- | --- | --- |
| ***Quadratopectoralis*** |  | *MQP* | ***Interhyoideus posterior*** *(Larsen & Guthrie, 1975) (Matsumoto, Fujiwara, & Evans, 2024) (Piatt, 1940),* ***superficial hyoid constrictor*** *(Lorenz Elwood & Cundall, 1994),* ***Interhyoideus*** *(Heiss, Handschuh, Aerts, & Van Wassenbergh, 2016) (Krogh & Tanner, 1972)* |
| ***Intermandibularis*** | *anterior + posterior* | *MIM* |  |
| ***Inter hyoideus*** | *anterior* | *MIH* | ***Interossaquadrata*** *(Heiss, Handschuh, Aerts, & Van Wassenbergh, 2016) (Krogh & Tanner, 1972),* ***interhyoideus anterior*** *(Piatt, 1940)* |
|  | *posterior* | *MIHp* | ***Quadratopectoralis*** *(Deban & Richardson, 2017) (Deban & Wake, 2000, a)* |
| ***Geniohyoideus*** |  | *MGH* | ***Coracomandibularis*** *(Deban & Wake, 2000, a),* ***geniohyoideus*** *(Larsen & Guthrie, 1975)* |
| ***Subarcualis rectus*** |  | *MSbR* | ***Ceratohyoideus*** *(Deban & Wake, 2000, a),* ***ceratohyoideus externus*** *(Deban & Wake, 2000, a)* |
| ***Subhyoideus*** |  | *MSbH* | ***Geniohyoideus lateralis*** *(Piatt, 1940)* |
| ***Branchiohyoideus*** |  | *MBH* | ***Branchiohyoideus externus*** *(Schwarz, Konow, Tolosa Roba, & Heiss, 2020)* *(Piekarski & Olsson, 2007)* |
| ***Genioglossus*** |  | *MGG* |  |
| ***Rectus cervicis*** | *Superficialis* | *MRCsup* | ***Stenohyoideus*** *(Deban & Wake, 2000, a) (Ziermann & Diogo, 2013) (Lauder & Bradley Shaffer, 1985)* |
|  | *Profondus* | *MRCpro* | ***Abdominohyoideus*** *(Deban & Wake, 2000, a)* |
| ***Depressor mandibulae*** | *Anterior* | *MDMa* |  |
|  | *Posterior* | *MDMp* |  |
| ***Adductor mandibulae externus*** |  | *MAME* | ***Levator mandibulae externus*** *(Deban & Richardson, 2017) (Deban & Wake, 2000, a) (Lorenz Elwood & Cundall, 1994) (Erdman & Cundall, 1984) (Krogh & Tanner, 1972) (Larsen & Guthrie, 1975) (Matsumoto, Fujiwara, & Evans, 2024) (Piatt, 1938) (Ericsson & Olsson, 2004) (Piekarski & Olsson, 2007),* ***adductor mandibulae externus complex*** *(Schwarz, Konow, Tolosa Roba, & Heiss, 2020),* ***adductor mandibulae A2*** *(Ziermann & Diogo, 2013)* |
| ***Adductor mandibulae internus*** | *Profondus (deep)* | *MAMIpro* | ***Pseudotemporalis profundus*** *(Carroll & Holmes, 1980) (Iordansky, 2010),* ***levator mandibulae internus anterior*** *(Deban & Richardson, 2017) (Deban & Wake, 2000, a),* ***deep levator mandibulae anterior*** *(Lorenz Elwood & Cundall, 1994) (Erdman & Cundall, 1984) (Hinderstein, 1971) (Krogh & Tanner, 1972) (Larsen & Guthrie, 1975) (Matsumoto, Fujiwara, & Evans, 2024),* ***pseudotemporalis profundus of the adductor mandibulae internus complex*** *(Schwarz, Konow, Tolosa Roba, & Heiss, 2020),* ***pseudotemporalis deep A3''*** *(Ziermann & Diogo, 2013) (Carroll, 2008)* |
|  | *Superficialis* | *MAMIsup* | ***Pseudotemporalis superficialis*** *(Carroll & Holmes, 1980) (Iordansky, 2010) (Ziermann & Diogo, 2013),* ***levator mandibulae internus posterior*** *(Deban & Richardson, 2017) (Deban & Wake, 2000, a),* ***superficial levator mandibulae anterior*** *(Lorenz Elwood & Cundall, 1994) (Erdman & Cundall, 1984) (Hinderstein, 1971) (Krogh & Tanner, 1972) (Larsen & Guthrie, 1975),* ***pseudotemporalis superficialis of the adductor mandibulae internus complex*** *(Schwarz, Konow, Tolosa Roba, & Heiss, 2020),* ***levator mandibulae anterior*** *(Piekarski & Olsson, 2007),* ***levator mandibulae anterior superficialis*** *(Piatt, 1938),* ***levator mandibulae longus*** *(Haas, 2001) (Ericsson & Olsson, 2004),* ***adductor mandibulae A3'*** *(Carroll, 2008)* |
|  | *Pterygoideus* | *MPt* | ***Pterygomandibularis*** *(Schwarz, Konow, Tolosa Roba, & Heiss, 2020)* |
| ***Adductor mandibulae posterior*** |  | *MAMP* | ***Levator mandibulae posterior*** *(Lorenz Elwood & Cundall, 1994) (Erdman & Cundall, 1984) (Krogh & Tanner, 1972) (Larsen & Guthrie, 1975) (Matsumoto, Fujiwara, & Evans, 2024),* ***Adductor mandibulae A2-PVM*** *(Ziermann & Diogo, 2013)* |

Supplementary Table 4: Shapiro-Wilk normality test statistics for all variables before and after log10-transformation. W: Shapiro-Wilk statistic; p‑value: significance of deviation from normality. Significant results are indicated in bold.

|  | **Before log10-transformation** | | **After log10-transformation** | |
| --- | --- | --- | --- | --- |
| **Variable** | **W statistic** | **p-value** | **W statistic** | **p-value** |
| **HL** | 0.871 | **0.003** | 0.945 | 0.157 |
| **Vol_HyoLev** | 0.741 | **0.001** | 0.956 | 0.303 |
| **Vol_HyoPro** | 0.484 | **0.001** | 0.957 | 0.318 |
| **Vol_HyoRet** | 0.385 | **0.001** | 0.932 | 0.075 |
| **Vol_MouthOp** | 0.586 | **0.001** | 0.933 | 0.080 |
| **Vol_MouthClo** | 0.576 | **0.001** | 0.964 | 0.465 |
| **pcsa_HyoLev** | 0.819 | **0.001** | 0.956 | 0.305 |
| **pcsa_HyoPro** | 0.488 | **0.001** | 0.955 | 0.285 |
| **pcsa_HyoRet** | 0.465 | **0.001** | 0.922 | **0.043** |
| **pcsa_MouthOp** | 0.602 | **0.001** | 0.975 | 0.742 |
| **pcsa_MouthClo** | 0.558 | **0.001** | 0.977 | 0.791 |

Supplementary Table 5: The table presents the estimates characteristics of the Volume and PCSA of the 4 models tested (Ornstein-Uhlenbeck, Pagel's lambda transformation, Brownian Motion, Early Burst), along with GIC and Log-likelihood values, for the 3 factors: ecological transition, morphological transformation and adult habitat.

|  | **Ornstein-Uhlenbeck** | | | |
| --- | --- | --- | --- | --- |
|  | **Volume** | | **PCSA** | |
|  | **GIC** | **Log-likelihood** | **GIC** | **Log-likelihood** |
| **Ecological transition** | 8.9 | 21.1 | 19.3 | 15.4 |
| **Morphological transformation** | 11.2 | 20.1 | 20.7 | 15.0 |
| **Adult habitat** | 4.4 | 24.9 | 8.4 | 23.1 |
|  | **Pagel's lambda** | | | |
|  | **Volume** | | **PCSA** | |
|  | **GIC** | **Log-likelihood** | **GIC** | **Log-likelihood** |
| **Ecological transition** | 14.3 | 19.1 | 25.0 | 13.3 |
| **Morphological transformation** | 14 | 19 | 25.7 | 13.1 |
| **Adult habitat** | 8.9 | 24.0 | 11.2 | 22.5 |
|  | **Brownian Motion** | | | |
|  | **Volume** | | **PCSA** | |
|  | **GIC** | **Log-likelihood** | **GIC** | **Log-likelihood** |
| **Ecological transition** | 46.4 | 2.9 | 59.2 | -4.2 |
| **Morphological transformation** | 41.3 | 5.1 | 54.3 | -2.4 |
| **Adult habitat** | 35.9 | 11.3 | 40.3 | 8.9 |
|  | **Early Burst** | | | |
|  | **Volume** | | **PCSA** | |
|  | **GIC** | **Log-likelihood** | **GIC** | **Log-likelihood** |
| **Ecological transition** | 48.4 | 2.9 | 61.2 | -4.3 |
| **Morphological transformation** | 43.3 | 5.1 | 56.3 | -2.4 |
| **Adult habitat** | 37.9 | 11.3 | 42.3 | 8.9 |

Supplementary Fig.1: Illustration of different functional muscle groups.

MAME (muscle adductor mandibulae externus), MAMIsup (muscle adductor mandibulae internus superficialis), MDM (muscle depressor mandibulae), MGH (muscle geniohyoideus), MGG (muscle genioglossus), MIH (muscle interhyoideus), MIM (muscle intermandibularis), MQP (muscle quadratopectoralis), MRCsup (muscle rectus cervicis superficialis), MSbR (muscle subarcualis rectus), and MSbH (muscle subhyoideus).


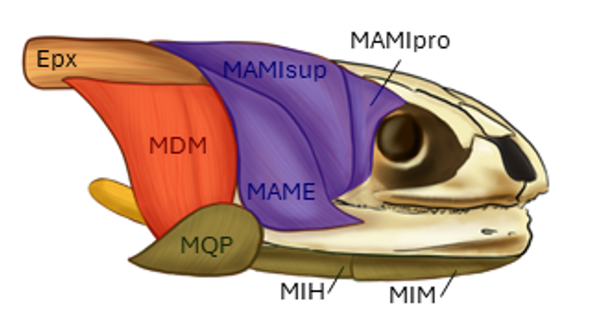

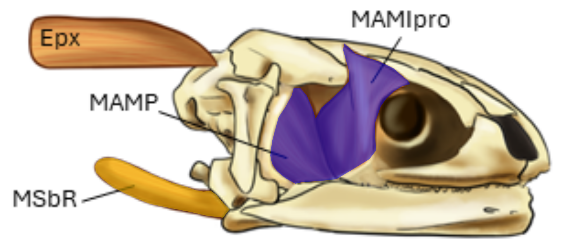

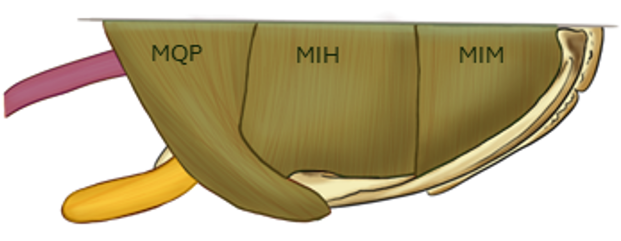

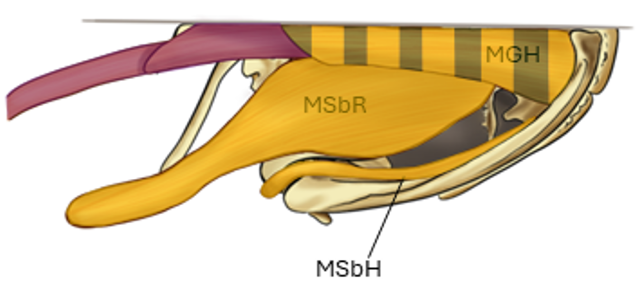

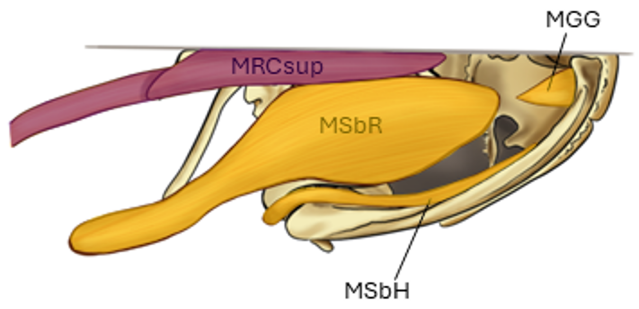

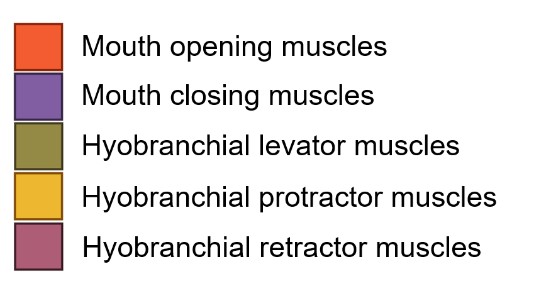


**References**

**Bauer W. J. (1997).** A contribution to the morphology of visceral jaw-opening muscles of urodeles (Amphibia: Caudata). Journal of morphology. 233(1). 77–97. [https://doi.org/10.1002/(SICI)1097-4687(199707)233:1<77::AID-JMOR7>3.0.CO;2-L](https://doi.org/10.1002/(SICI)1097-4687(199707)233:1%3c77::AID-JMOR7%3e3.0.CO;2-L)

**Carroll. R. L.. & Holmes. R. (1980).** The skull and jaw musculature as guides to the ancestry of salamanders. Zoological Journal of the Linnean Society. 68(1). 1-40. <https://doi.org/10.1111/j.1096-3642.1980.tb01916.x>

**Deban. S. M.. & Richardson. J. C. (2017).** A peculiar mechanism of bite-force enhancement in lungless salamanders revealed by a new geometric method for modeling muscle moments. Journal of Experimental Biology. 220(19). 3588-3597. <https://doi.org/10.1242/jeb.165266>

**Deban. S. M.. & Wake. D. B. (2000 a).** Aquatic feeding in salamanders. In K. Schwenk (Ed.). Feeding (pp. 65-94). Academic Press. <https://doi.org/10.1016/B978-012632590-4/50004-6>

**Erdman. S.. & Cundall. D. (1984).** The feeding apparatus of the salamander Amphiuma tridactylum: Morphology and behavior. Journal of morphology. 181(2). 175–204. <https://doi.org/10.1002/jmor.1051810206>

**Ericsson. R.. & Olsson. L. (2004).** Patterns of spatial and temporal visceral arch muscle development in the Mexican axolotl (Ambystoma mexicanum). Journal of Morphology. 261. 131–140. <https://doi.org/10.1002/jmor.10151>

**Haas. A. (2001).** Mandibular arch musculature of anuran tadpoles. with comments on homologies of amphibian jaw muscles. Journal of Morphology. 247(1). 1–33. [https://doi.org/10.1002/1097-4687(200101)247:1<1::AID-JMOR1000>3.0.CO;2-3](https://doi.org/10.1002/1097-4687(200101)247:1%3c1::AID-JMOR1000%3e3.0.CO;2-3)

**Heiss. E.. Handschuh. S.. Aerts. P.. & Van Wassenbergh. S. (2016).** Musculoskeletal architecture of the prey capture apparatus in salamandrid newts with multiphasic lifestyle: does anatomy change during the seasonal habitat switches? Journal of Anatomy. 228(5). 757-770. <https://doi.org/10.1111/joa.12445>

**Hinderstein. B. (1971).** The Desmognathine Jaw Mechanism (Amphibia: Caudata: Plethodontidae). Herpetologica. 27(4). 467-476.

**Iordansky. N. N. (2010).** Pterygoideus muscles and other jaw adductors in amphibians and reptiles. Biology Bulletin. 37(9). 905-914. <https://doi.org/10.1134/S1062359010090050>

**Krogh. J. E.. & Tanner. W. W. (1972).** The hyobranchium and throat myology of the adult Ambystomidae of the United States and northern Mexico. Brigham Young University Science Bulletin. Biological Series. 16(1). 1-69.

**Larsen. J. H.. & Guthrie. D. J. (1975).** The feeding system of terrestrial tiger salamanders (Ambystoma tigrinum melanostictum baird). Journal of Morphology. 147(2). 137-153. <https://doi.org/10.1002/jmor.1051470203>

**Lauder. G. V.. & Bradley Shaffer. H. (1985).** Functional Morphology of the Feeding Mechanism in Aquatic. Journal of Morphology. 185(5). 297-326. <https://doi.org/10.1002/jmor.1051850304>

**Lorenz Elwood. J. R.. & Cundall. D. (1994).** Morphology and behavior of the feeding apparatus in Cryptobranchus alleganiensis (Amphibia: Caudata). Journal of Morphology. 220(1). 47-70. <https://doi.org/10.1002/jmor.1052200106>

**Matsumoto. R.. Fujiwara. S.-I.. & Evans. S. S. (2024).** The anatomy and feeding mechanism of the Japanese giant salamander (Andrias japonicus). Journal of anatomy. 244(5). 679-707. <https://doi.org/10.1111/joa.14004>

**Piatt. J. (1938).** Morphogenesis of the cranial muscles of Amblystoma punctatum. Journal of Morphology. 63(3). 531–587. <https://doi.org/10.1002/jmor.1050630306>

**Piatt. J. (1940).** Correct Terminology in Salamander Myology II. Transverse Ventral Throat Musculature. Copeia. 1940(1). 9–14. <https://doi.org/10.2307/1439016>

**Piekarski. N.. & Olsson. L. (2007).** Muscular derivatives of the cranialmost somites revealed by long-term fate mapping in the Mexican axolotl (Ambystoma mexicanum). Evolution & Development. 9(6). 566–578. <https://doi.org/10.1111/j.1525-142X.2007.00197.x>

**Schwarz. D.. Konow. N.. Tolosa Roba. Y.. & Heiss. E. (2020).** A salamander that chews using complex. three-dimensional. Journal of Experimental Biology. 223(5). <https://doi.org/10.1242/jeb.220749>

**Ziermann. J. M.. & Diogo. R. (2013).** Cranial muscle development in the model organism Ambystoma mexicanum: Implications for tetrapod and vertebrate comparative and evolutionary morphology and notes on ontogeny and phylogeny. The Anatomical Record. 296(7). 1031-1048. <https://doi.org/10.1002/ar.22713>
